# A Semi-quantitative Evaluation of Out-to-Out Agenesis of Posterior Wall in a Dry Human Sacrum in Bangladesh

**DOI:** 10.7759/cureus.31163

**Published:** 2022-11-06

**Authors:** Rawshon Ara Naznin, Md. Ahsanul Haq, Sharmin A Sumi, Rahnuma Ahmad, Mainul Haque

**Affiliations:** 1 Anatomy, TMSS (Thengamara Mohila Sabuj Sangha) Medical College, Bogra, BGD; 2 Bio-Statistics, Infectious Diseases Division, icddr,b, Dhaka, BGD; 3 Anatomy, Bangabandhu Sheikh Mujib Medical University (BSMMU), Dhaka, BGD; 4 Physiology, Medical College for Women and Hospital, Dhaka, BGD; 5 Pharmacology and Therapeutics, National Defence University of Malaysia, Kuala Lumpur, MYS

**Keywords:** embryology, genetic mutation, congenital anomaly, caudal block, spina bifida, agenesis, sacral canal, sacral foramina, sacrum, sacral vertebrae

## Abstract

Background: Each vertebra is formed by combining the distal portion of one somite and the cranial half of its proximate part. *HOX* genes regulate the patterning of the shapes of the non-identical spinal column. In the sacral area, anatomical dissimilarity is on account of the distinct shape of the sacral hiatus and the outright non-subsistence of the posticous embankment of the sacral neural tube, which is a consequence of the non-fulfillment of bonding of lamina of all sacral vertebrae. After that, the meninges and the spinal nerve are unprotected and undiagnosable without an X-ray examination. Therefore, it is difficult to detect the reasons for caudal block failure, low back pain, etc. The current research aimed to improve the proficiency of anatomical and developmental errors of the dorsal wall of the sacrum.

Methods: This study was conducted on 60 dried adult sacra of unknown sexes from the stock wing of Anatomy, Sylhet MAG Osmani Medical College, Bangladesh, from 2017 to 2018. The undefined gender of the sacrum was identified.

Results: Out of 60 sacra, 30 (50.0%) were found to be that of males and 30 (50.0%) of females. Among the study samples, only three (5%) samples presented a complete absence of the sacrum's dorsal wall and and incidence among males was higher than females.

Conclusion: This type of sacral aberration has paramount clinical importance. Thereby avoiding caudal epidural block-connected sufferings and backbone operative procedures. The expertise regarding the anatomical variation of sacral hiatus is necessary to reduce the failure rate during caudal epidural anesthesia, helps orthopedic surgeons diagnose the cause of low back pain or in surgical situations, and helps pediatricians deal with congenital anomalies such as meningocele and myelomeningocele.

## Introduction

Anatomy

Among the 33 vertebrae, the sacrum consists of five sacral vertebrae located in the rear part of the human spinal column [1]. The ventral fence of the sacrum contains four pairs of ventral sacral foramina, which pass on the forward-facing ramus of S1-S4 vertebral cord nerves. The dorsal wall of the sacrum also includes four pairs of dorsal sacral foramina, which transmit dorsal ramus of S1-S4 spinal nerves [2], and the sacral tube (duct) is the prolongation of the vertebral channel. The vertebral, a tubular anatomical passage contains the spinal cord that terminates in the neighborhood of the lumbar area (L1-L2); the dura mater reaches out to S2 [3]. The sacral tube or duct is completed as sacral hiatus, configurated on account of the non-fulfillment of the bonding of lamina of the fifth sacral vertebra. So, sacral canal comprises sacral nerves, filum terminale [4]. Sacral hiatus transmits a couple of the fifth sacral nerves, a duplet of coccygeal nerves, filum terminale externa, fibrofatty tissue, and ligamentum flavuma [5,6].

The sacrum's caudal part is articulated with the coccyx and filum terminal end by blending with the coccyx's dorsal aspect [7,8]. The neurovascular structure is a very important anatomical structure. Usually, it is in the dorsal wall of the sacrum. Generally, its dorsal wall is convex from the sagittal view. The median sacral crest (fused rudimentary sacral spinous processes) is constructed by the merging posterior constituents of the sacral vertebrae. The coalesced laminae form sacral grooves; the merged articular processes configure the intermediary sacral crest, and the meld transverse processes shape the lateral sacral crests. Typically, posterior sacral neuroforamina is four pairs and sprawls beside the lateral to the intermediate sacral crests. Superior articular processes are the superior continuation of intermediate sacral crests of the S1 vertebra combined with the L5 vertebra. On each side of the sacral hiatus, there are palpable prominences acknowledged as sacral cornua, which are located backwardly to the inferior articular processes of S5. Beyond the inferior portion of the S4, the median sacral topknot and trough are deficient and form the sacral hiatus [9], which is covered by a connective tissue structure known as the sacrococcygeal membrane. Many variations are present in the sacral canal, located in the dorsal wall, and may be low-lying or open throughout its entire length [10]. In addition to sacral agenesis (SA), several malformations occur in the caudal region, known as the syndrome of caudal regression [11].

Embryology and genetics

The cardiovascular and musculoskeletal systems develop from the mesoderm of the embryo. Mesoderm arises from the primitive streak (PS) during gastrulation (the early developmental stage of the human embryo). A high bone morphogenetic protein (BMP) at the rearward of PS sows the seeds of ventral mesoderm (blood vessels, lateral and extraembryonic mesoderm). The anterior extreme sharp end of the PS suffers from bone morphogenic protein that causes paraxial mesoderm. This paraxial mesoderm is responsible for somites formation, thereby negatively affecting the development of midline striated muscle and axial skeleton [12,13]. The sclerotome portion of the somites gives rise to essential portions related to vertebrae [14-16]. Sacral hiatus has a different shape due to the notochord influence's defective triggering process of vertebra genesis. This process was initiated in the time of embryological development and probably through altered sonic hedgehog genetic impact [17]. *HOX* genes 5 regulate the tessellated configuration of the divergent vertebrae column [18,19]. However, one’s own alteration in genetic elements such as *VANGL1* [20], *HOXD13* [21], and *PTEN* [22] have been observed.

Additionally, an absence of a particular structure is called agenesis. If agenesis is observed on the sacrum's dorsal wall, it is called spina bifida. Spina bifida formation is possibly related to a transmutation in the *VANGL* and *HOX* genes. *VANGL* genes are a fragment of the planar cell polarity route that controls the lengthening unification procedure. This is essential for the typical neural tube elongation and shutdown process. Homeobox gene codes for transliteration elements instigate the outpouring of genes controlling episodes for segmentation and axis development [20,23,24].

Different shapes of sacral hiatus with their importance

Different authors have found different shapes during their research work. Bagoji et al. found various sacral hiatus shapes such as inverted U, inverted V, dumbbell, irregular, and bifid [25]. Vasuki et al., Lees et al., and Bagheri et al. revealed similar findings [26-28]. Kamal et al. reported two additional shapes such as rough and elongated [29]. There is no exact importance of the different forms of sacral hiatus. Furthermore, Abera et al. suggested a substantial proportion of abnormal extension and narrow width of sacral hiatus [30]. Extended and restricted sacral hiatus are important clinical issues while conducting the caudal anesthetic procedure. On the contrary, Mustafa et al. suggested putting a required surgical instrument into the sacral hiatus for the caudal anesthetic procedure by regulation conducted through the bottom to avert the anatomic inequality of the tip of the sacral hiatus [31].

Clinical importance of neural tube defects

When the neural tube does not close completely, it is known as a neural tube defect (NTD), which represents a group of disorders (such as spina bifida and anencephaly) that affect the evolution of the central nervous system [32]. The severe complications of NTDs include stillbirth and preterm labor leading to abortion [33]. NTDs may occur anywhere in the vertebral column. Many factors influence the development of NTDs, such as genetic factors, environmental factors, obesity, diabetes, deregulated immune function, folic acid antagonists, dihydrofolate reductase inhibitors, socioeconomic level, earth science, ethnic background, amniotic bands, and hyperthermia during pregnancy. Also, radiation, stress, hypervitaminosis A, rubella, toxoplasmosis, and cytomegalovirus acts as physical and chemical environmental factors [34-36].

Spina bifida can be recognized during pregnancy through ultrasonography to pinpoint the precise measurements and position of neural tube deformity and vertebrae. Additionally, the quantity of α-fetoprotein in maternal serum and amniotic fluid can be assessed as a high α-fetoprotein level supports the presence of NTDs. α-fetoprotein is manufactured by the fetal yolk sac, liver, and alimentary canal [37,38]. At the same time, we can evaluate the anomaly more accurately by using magnetic resonance imaging (MRI) [39]. If we want to see the relationship between genetic mutation and NTD, we can do a chromosomal microarray [40].

The variations of spina bifida are: (i) Spina bifida occulta, which is an anomaly in the vertebral arches that are shielded/protected by skin. Typically, in this vertebral deformity, neural tissue physiology remains normal. Spina bifida occulta is found in the lumbosacral area (L4-S1) and is generally manifest by a stripe of hair carpeting the concerned locale. It has been observed in around 10% of some otherwise healthy individuals; (ii) Spina bifida cystica, which is a grave NTD that affects neural tissue and/or meninges. These neural structures come through vertebral arches and skin to construct resembling a fluid-containing bag. Most of these anatomical anomalies are situated in the lumbosacral areas with the neurologic shortfall. This can also be of two types: (a) Spina bifida with meningocele, which contains transparent watery liquid that sticks out through the gap formed due to a development error of the sacral lamina, and (b) Spina bifida with meningomyelocele, which contains additionally neural tissue and clear watery liquid covered with meninges that comes out through the anatomical, developmental anomaly defect; and (iii) Spina bifida with myeloschisis or rachischisis in which, at times, the neural folds do not protrude but remain as a demolished and compressed heap of neural tissue [41].

Comprehensive proficiency in sacral anatomical and developmental variances is crucial for varied medical professionals such as orthopedic surgeons, neurosurgeons, neurologists, urologists, anesthesiologists, obstetricians, radiologists, and forensic pathologists. Furthermore, it has been reported that appropriate knowledge regarding congenital anomalies is essential for surgical experts dealing with these genetic errors in order to achieve the best clinical outcome [42-44]. So, accurately identifying defective sacral canal walls can help avoid complications in treating patients [45,46].

Spina bifida and caudal nerve block

Caudal epidural blocks are often appraised as free from hazard and an effective method of perioperative analgesia, especially for infants and neonates for minor surgical procedures, including circumcision [47,48]. The sacrum is the clinically all-important anatomical area for the caudal epidural anesthetic procedure. These anesthetic approaches are frequently conducted for diagnostic and treatment purposes for lumbosacral disease management. Caudal anesthesia is given in a range of surgical procedures such as inguinal or umbilical hernias, hydrocoele, orchidopexy and hypospadias, circumcision, and anorectal and genitourinary surgery [49-51]. In case of unusual sacral hiatus extension or agenesis of the sacral canal's posterior fence, it will be troublesome to spot the landmark, leading to caudal anesthetic failure [52]. This route also gives children postoperative analgesia [47,53]. There may be the risk of puncturing the dural sac or inadequate pain minimization due to sacral anatomical anomalies [54].

Though there are many anatomical variants of the sacrum at the dorsal wall, knowledge about the anatomy and anatomical variation [55-57] of the dorsal wall of the sacrum is fundamental in many clinical situations, including the most vital issue of human life, spina bifida.

The objective of the study

To the authors' best knowledge, this is the first study on abnormal sacral canal conditions in Bangladesh. The present study was conducted to ascertain the anatomical anomalies of the dorsal wall of the sacrum by way of morphometric evaluation among Bangladeshi people. This study will possibly help different populations and geographical areas to successfully deal with ailments related to varying shapes of sacral hiatus or absence of form, comparing data in these populations with those of the world and enhancing the repository of anatomical and developmental errors of the sacrum.

## Materials and methods

This study followed the observational research method. The study specimens were obtained from the Department of Anatomy, Sylhet MAG Osmani Medical College, Sylhet, Bangladesh. More than 60 dried sacra of unknown sex were randomly selected for the study. The opportunity sampling method was adopted to select sacrum; thereby, we took the same sample size for both sexes for an appropriate comparison among the sexes. Through a visual examination and sacral indexing, a total of 30 male and 30 female sacra were retained for the study purpose. Completely dried grossly normal adult sacra were included in this study, which was conducted from July 2017 to June 2018. Ethical approval was received from the Institutional Review Board, Sylhet MAG Osmani Medical College, Sylhet, Bangladesh (Approval number: SOMC/2018/298, dated March 24, 2018).

Data analysis

The data was initially hand-computed in a predesigned Excel spreadsheet template (Microsoft Corporation, Redmond, Washingtion, United States). Data were described using mean with standard deviation (SD). Independent sample t-test was used to assess the mean difference in sacral index percentage between sexes. An independent t-test is a statistical test to detect the significance level. As the sample size n>30, we could perform the independent sample t-test. Nonetheless, the normality plot as well as the skewness (p=0.607) and Kurtosis (p=0.181) indicated the data were normally distributed. We also used univariate regression to see the mean difference of non-identical configuration of sacral foramen with sacral index percentage. The significance level was established as p ≤ 0.05. The statistical analyses were performed with Stata Statistical Software: Release 15 (StataCorp LLC, College Station, Texas, United States) and IBM SPSS Statistics for Windows, Version 22.0 (Released2913; IBM Corp., Armonk, New York, United States), and the graphs were prepared by GraphPad Prism 8.3.0 (Dotmatics, Boston, Massachusetts, United States).

## Results

**Figure 1:**
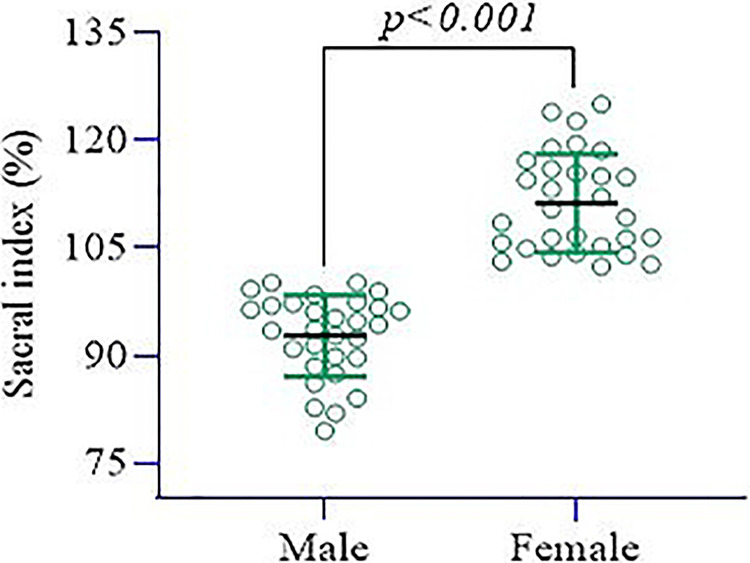
Independent sample t-test showed that the sacral index percentage was significantly higher in females (mean±sd) 111.2±6.88 compared to males (92.7±5.68) p<0.001 Image Credit: Md Ahsanul Haq

The total sample size was 60, with 30 males (92.7±5.68) and 30 females (111.2±6.88), according to the sacral index that was statistically significant (p<0.001) (Figure 1).

**Figure 2:**
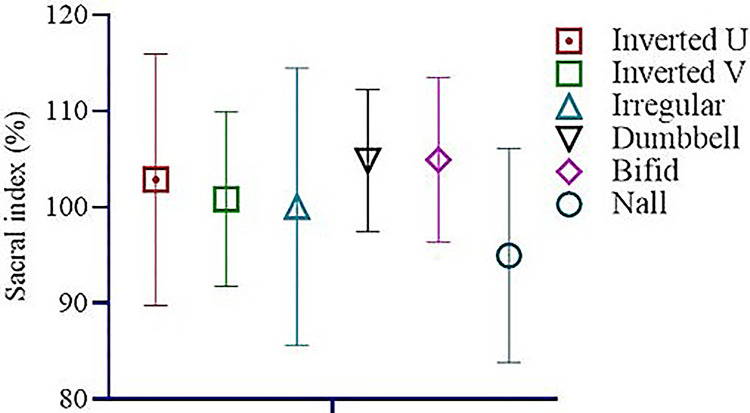
Univariate regression was used to see the mean difference of non-identical configuration of sacral foramen with sacral index percentage. No significant difference was noted Null=Agenesis Image Credit: Md Ahsanul Haq

**Figure 3:**
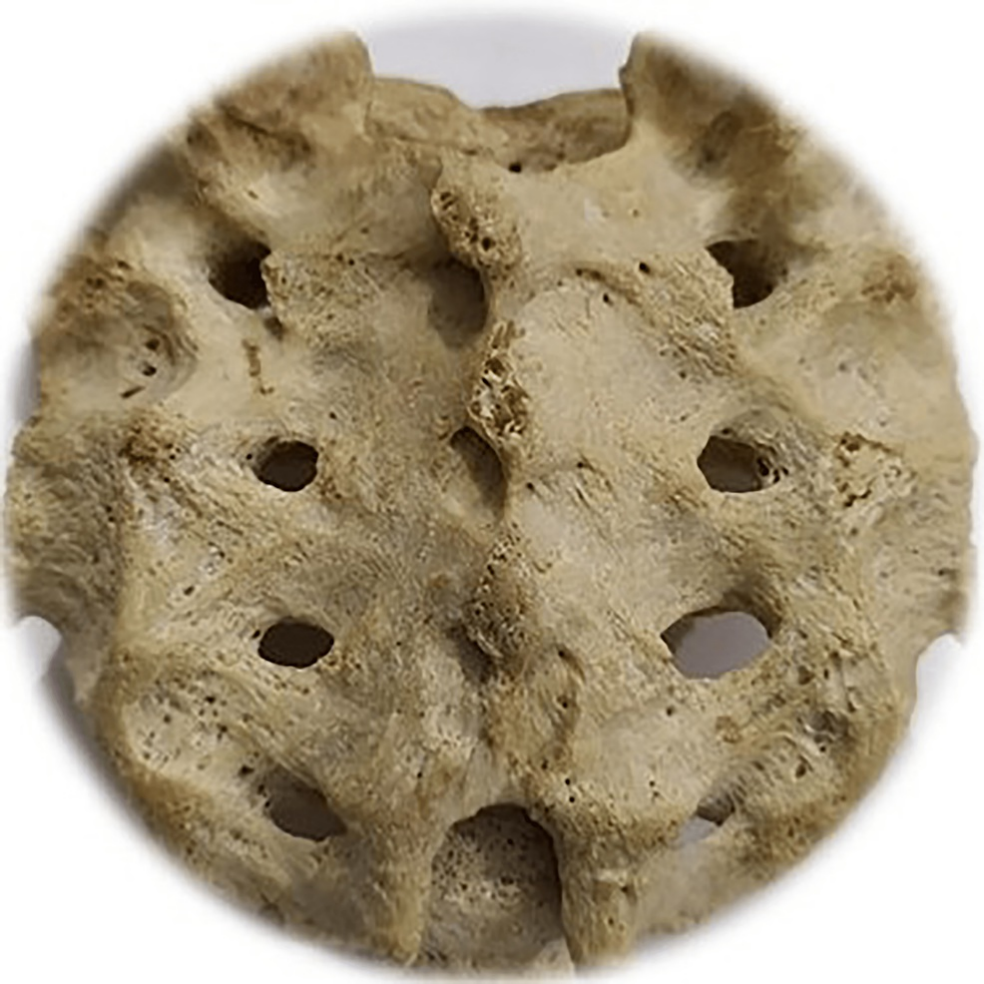
Sacral hiatus with inverted U structure Photo Credit: Rawshon Ara Naznin

**Figure 4:**
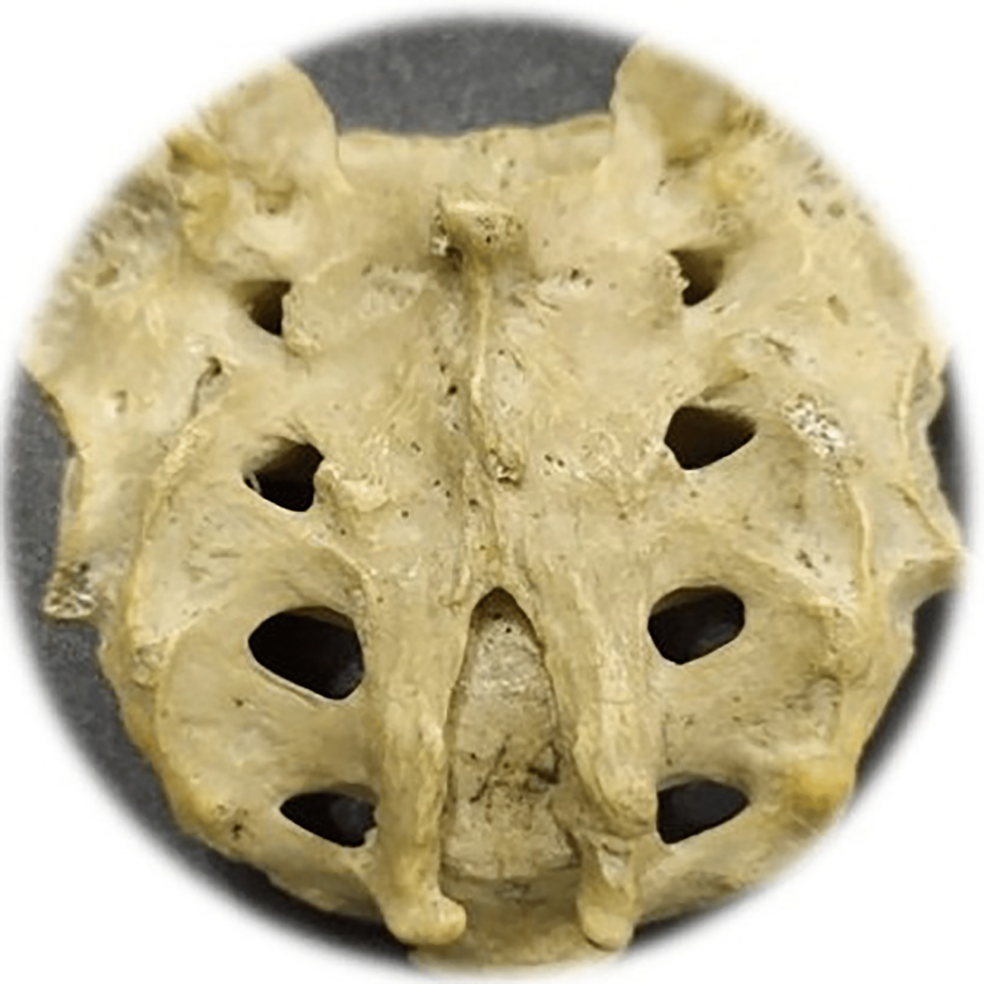
Sacral hiatus with inverted V form Photo Credit: Rawshon Ara Naznin

**Figure 5:**
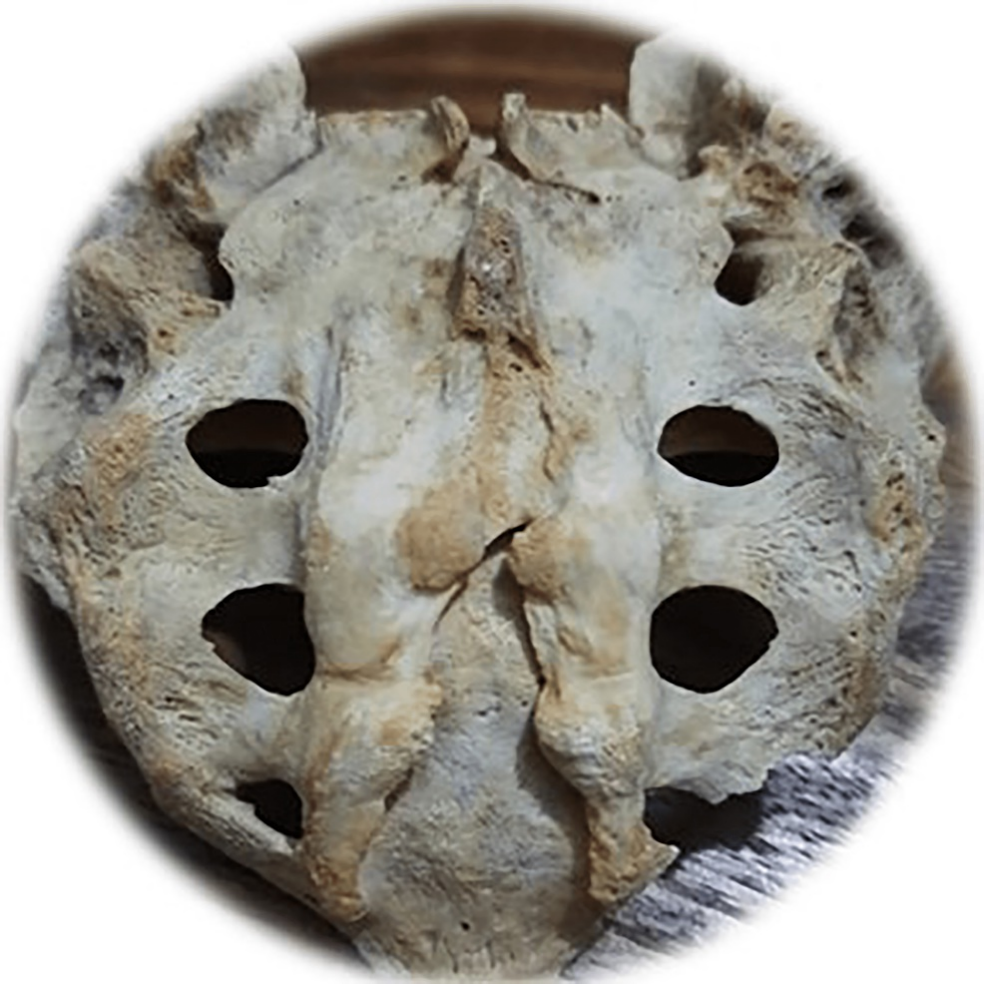
Sacral hiatus with irregular appearance Photo Credit: Rawshon Ara Naznin

**Figure 6:**
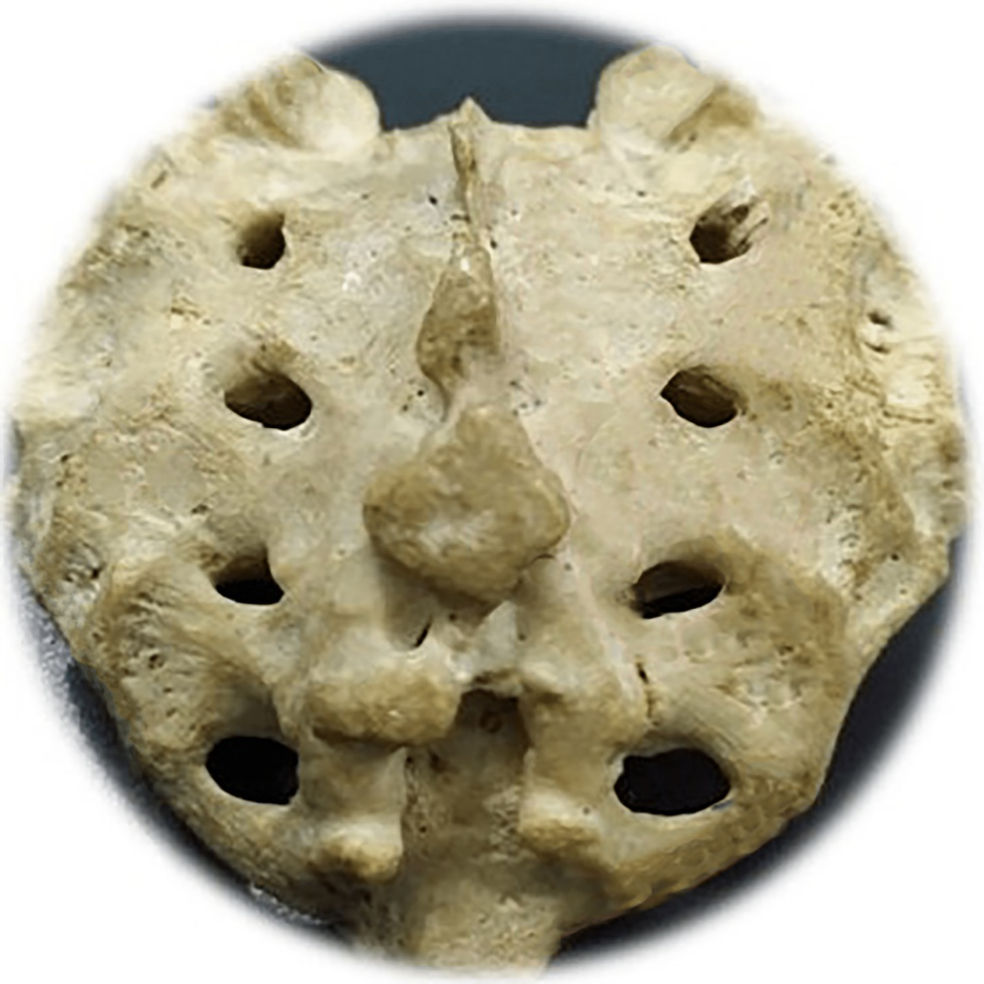
Sacral hiatus with dumbbell shape Photo Credit: Rawshon Ara Naznin

**Figure 7:**
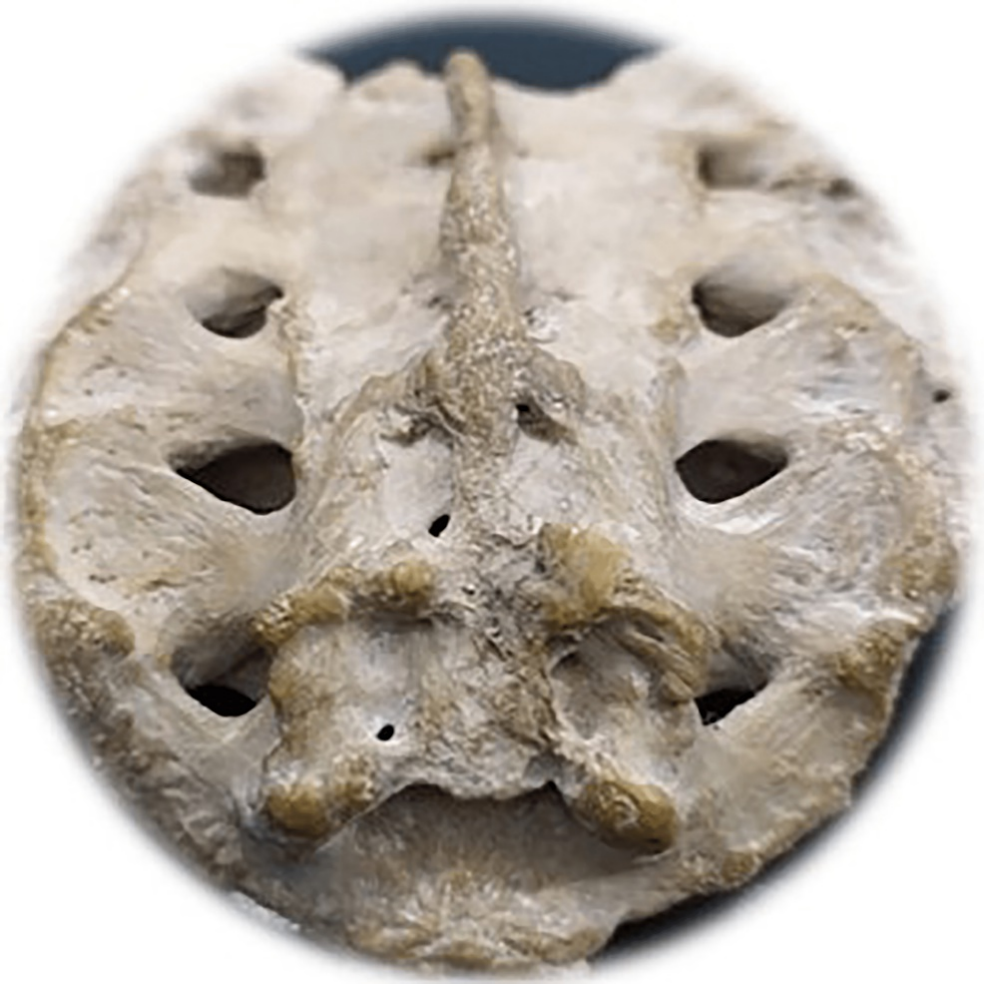
Sacral hiatus showing bifid build Photo Credit: Rawshon Ara Naznin

**Figure 8:**
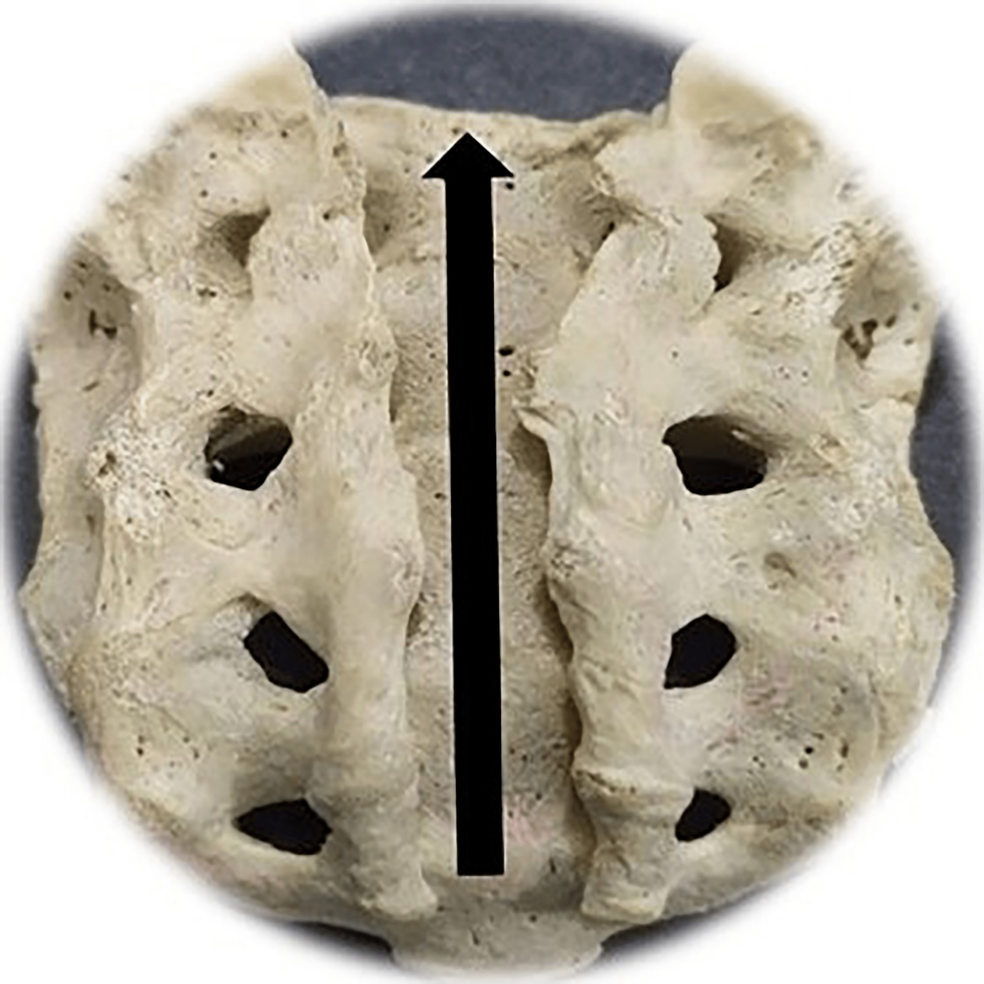
Total agenesis of the posterior fence of the sacral canal Photo Credit: Rawshon Ara Naznin

**Figure 9:**
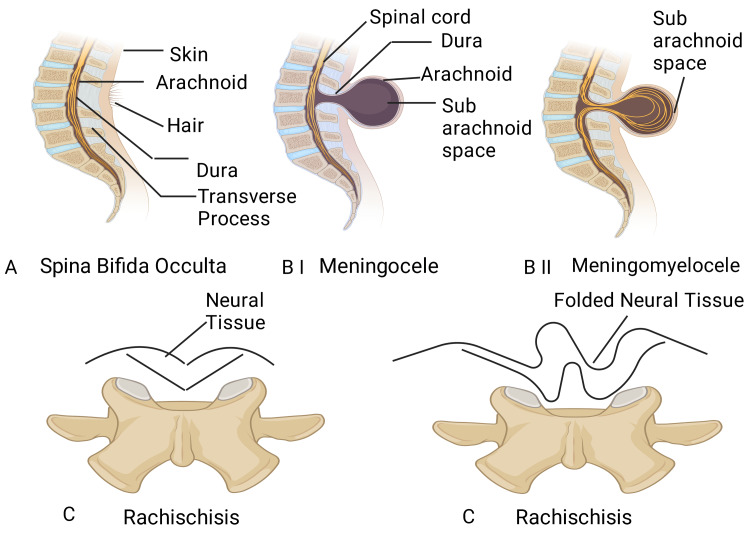
The different types of spina bifida Image Credit: Rahnuma Ahmad; this figure has been developed using BioRender (https://biorender.com/), license number: QP24KSKKPO

Among the study sample, 57 of the sacra (Table 1) were the exact shape of sacral hiatus. Univariate regression analysis was conducted between non-identical sacral configuration and sacral index, which revealed no significant (p>0.05) difference (Figure 2). This study found six different types of sacral shapes. Those were inverted U (n=24) (102.87±13.1) (Figure 3), inverted V (n-16) (100.85±9.07) (Figure 4), irregular (n=7) (100.08±14.4) (Figure 5), dumbbell (n=7) (104.84±7.41) (Figure 6), bifid (n=3) (104.94±8.59) (Figure 7). Only three (95.00±11.2) sacra showed agenesis (nonappearance) of the vertebra's hindmost barrier or fence of the sacral tube (Figure 8). Among the sacra showing agenesis, two (3.33%) were male and one (1.67%) was female. The types of agenesis are depicted in Figure 9.

**Table 1:** Mean difference of different shapes of sacral hiatus with sacral index percentage

The shape of the sacral hiatus	Sacral Index % (mean±SD)
Inverted U (n=24)	102.87±13.1
Inverted V (n=16)	100.85±9.07
Irregular (n=7)	100.08±14.4
Dumbbell (n=7)	104.84±7.41
Bifid (n=3)	104.94±8.59
Nall (n=3)	95.00±11.2

## Discussion

We examined completely ossified adult human sacra sample of anonymous sex in the current study, 50% of which were male and 50% female. This study found an inverted U in the shape of sacral hiatus (40%), which was almost similar to one earlier Indian study (42.02%) [25]. However, the current study findings were lower than one earlier study conducted in the United Arab Emirates (UAE) (56%) [58]. The inverted V shape (27%) of the current study was similar to another Indian research (27.51%) [59]. However, in an Indian study by Kumar et al. [60] and an Ethiopian study by Abera et al. [30], the most recurrently found sacral hiatus was inverted V (76.23% and 41%, respectively). Our study found the irregular shape to be 11.5%, which was lower than studies in India conducted by Sema et al. (16.10%) [59] and David (23.30%) [61]. The dumbbell shape was 11.5% in the current study, which was in the same line as Sema et al. (11.40%) [59]. One earlier study from Bangladesh reported dumbbell shape of 13.3% [29]. The bifid shape in our study was 5.0%, but other studies reported lower numbers (1.5%) [29]. Furthermore, Bagoji et al. reported almost similar findings (5.07%) [25].

The total absence (agenesis) of the sacral canal of the dorsal wall among the Bangladeshi population was 5.0%. Multiple studies revealed that the incidence of complete agenesis of the dorsal wall in the sacrum varies from 2.58% to 20% (Table 2) [10,30,59,62-69]. In our study, sacral agenesis (SA) was higher in males than females. Multiple studies reported an incidence of SA in 0.01-0.05/1000 live births [70-75]. This congenital anomaly is said to be of unidentified etiology; nevertheless, the potential contribution of genetic and teratogenic components prevails [76]. Another study revealed that SA among infants was commonly observed in diabetic mothers. It has also been reported that severe forms of cardiac, renal, and respiratory congenital anomalies exist with SA and cause a high newborn death rate [77]. Another study reported SA also exists with anorectal, urogenital deformation, and a presacral heap [78]. One more research revealed that orthopedic and neurological co-occurring diseases are time after time linked with SA and are more ubiquitous with myelomeningocele [79]. Meningocele is another frequent SA disorder of congenital origin often found with orthopedic, spinal, abdominal, and thoracic vital structure malformations [73]. Early therapeutic intervention, especially interdisciplinary management with the surgical course of action, frequently reduces SA-related morbidity and prevents mortality [80,81]. NTD necessitates diverse pediatric surgical doctors. Those include surgery, neurosurgery, orthopedics, urology, and nephrology for appropriate surgical and medical intervention to reduce morbidness and fatality [82,83]. 

**Table 2:** Comparison of incidence of complete dorsal wall agenesis by different authors

Serial No.	Author (Year of Study)		No. of Specimen Studied	Incidence of complete dorsal wall agenesis (%)
1.	Yonguc et al. [62] (2013)		110	20%
2.	Sema et al [59] (2013)		159	3.14%
3.	Nagendrappa and Jayanthi [63] (2014)		100	3%
4.	Mishra et al. [64] (2014)		93	4.3%
5.	Malarvani et al. [10] (2015)		100	3%
6.	Akhtar et al. [65] (2015)		116	2.58%
7.	Abera et al. [30] (2021)		61	3.3%
8.		Vanitha et al. [66] (2014)	Single Case Report	
9.		Gaikwad et al. [67] (2019)	Single Case Report	
10.		Swathi [68] (2013)	Single Case Report	
11		Aragao et al. [69] (2019)	45	4.4%
12.		Present study	60	5.00%

Preventive strategies

Parental environmental factors such as aluminum, cobalt, chromium, iron, selenium, and vanadium are often related to NTD [84]. Therefore, it reduces children's and adults' morbidity and mortality rate. It has been revealed that fortification of staple foods with folic acid after assessing low folate status among community eating habits. Consequently, it reduces folic acid deficiency and NTDs [85]. In Bangladesh, tubewell water often contains high amounts of inorganic arsenic. Therefore, drinking tubewell water and folic acid supplementation may help prevent NTDs [86].

Limitations of this study

This study sample size was small, only 60. If it were more extensive, the results would be more accurate. Time and financial constraints were principal obstacles in conducting this study in multiple centers.

## Conclusions

Awareness about the imperfect development of the posterior wall of the sacral tube is essential for radiologists, neurologists, anesthesiologists, pediatric surgeons, obstetricians, and orthopedics to manage clinical disorders that need regional block and low back pain. Inherited abnormality, for instance, the lumbosacral transitional vertebrae and the out-and-out lack or failure of development of the rearward wall, may confuse the differential diagnosis or endanger the surgical result if not considered. So, understanding this variation may decrease the failure rate of caudal epidural anesthesia and minimize complications during surgeries. Additional large-scale multicenter studies of the dorsal wall of the sacral canal of human desiccated sacrum are recommended, as the present study was conducted in a limited territory. 
